# ER morphological analysis associated with interstitial cells of Cajal and smooth muscle cells in the murine stomach

**DOI:** 10.1007/s00441-025-04016-7

**Published:** 2025-10-28

**Authors:** Hiromi Tamada, Satoshi Iino

**Affiliations:** Anatomy, Graduate School of Medical Sciences, University of Fukui, 23-3 Matsuokashimoaizuki, Yoshida-Gun, Eiheiji-cho, Fukui 910-1193 Japan

**Keywords:** Interstitial cells of Cajal, Endoplasmic reticulum, Focused ion beam/scanning electron microscopy, Plasma membrane

## Abstract

In the gastrointestinal tract, several modulators are involved in generating complex motility patterns, including smooth muscle cells (SMCs), interstitial cells of Cajal (ICCs), and PDGFRα+ cells. Notably, ICCs are excitable cells that generate characteristic oscillations in pacemaker activity mediated by the cytoplasmic Ca^2+^ concentration. Since the primary source of cytoplasmic Ca^2+^ is the endoplasmic reticulum (ER) or sarcoplasmic reticulum (SR), its distribution and localisation in the cytoplasm may indicate characteristic aspects that help in understanding its physiological functions. Furthermore, caveolae, which are invaginations of the plasma membrane (PM) observed in both ICCs and SMCs, are key sites for Ca^2+^ sparks, and their functions have been well studied in muscle cells. However, the mechanism by which caveolae and ER interact to regulate Ca^2+^ in ICCs remains unclear compared with that in SMCs. In this study, microdomains comprising the PM, caveolae, and ER/SR were analysed using focused ion beam/scanning electron microscopy (FIB/SEM) to provide new insights into ICC functional analysis. Additionally, as mitochondria could regulate local Ca^2+^ concentration, mitochondria-associated membrane (MAM) contacts with the ER were also analysed in ICCs. Novel ER ultrastructures with distinct characteristics and distributions were identified in each cell type by FIB/SEM. Furthermore, reconstructed three-dimensional (3D) images enabled measurement of the distance between the PM and ER, as well as the MAM areas, thus contributing to a better understanding of ICC physiological features. These new morphological insights may help resolve controversial interpretations in physiological and pharmacological studies of ICCs.

**Supplementary Information:**

The online version contains supplementary material available at 10.1007/s00441-025-04016-7.

## Introduction

Several cell types in the gastrointestinal tract contribute to the generation and coordination of gut motility. Smooth muscle cells (SMCs) are primarily responsible for motility, whereas interstitial cells of Cajal (ICCs) and PDGFRα+ cells are involved in coordinating motility. Similar to other muscle types, such as skeletal and cardiac muscle cells (Berridge 2006), smooth muscles require Ca^2+^ oscillations for contraction. ICCs are highly excitable cells that generate spontaneous pacemaker potentials, and some act as mediators of excitatory neurotransmission (Gomez-Pinilla et al. 2009; Sanders et al. 2024). PDGFRα+ cells, previously referred to as fibroblast-like cells, have been well studied as intermediators for inhibitory neurotransmission (Iino and Nojyo 2009; Kurahashi et al. 2011, 2013, 2014, and 2022). For these regulatory cells, Ca^2+^-dependent conductance is crucial for function. Because the endoplasmic reticulum (ER) and sarcoplasmic reticulum (SR) are among the most important sources or regulators of cytoplasmic Ca^2+^ (Zhu et al. 2009; Hwang et al. 2009; Klein et al. 2013; Malysz et al. 2017; Drumm et al. 2018), the structure and distribution of the ER/SR are likely to be key factors influencing their physiological functions in addition to the expression of ion channels and regulatory proteins. Although these cells are well characterised as ER/SR-rich cells when observed by transmission electron microscopy (TEM) (Komuro et al. 1999; Ishikawa and Komuro 1996), there is no information available on the extent to which ER/SR structures are similar or different at the three-dimensional (3D) level, for example, whether they are mesh- or tubular-like structures (Elgendy et al. 2024).

Additionally, when considering the role of Ca^2+^ in ICCs and SMCs, the caveolae on the plasma membrane are prominent structural features. Their presence is one of the criteria for detecting ICCs using TEM (Tamada and Komuro 2012; Komuro et al. 1999; Ishikawa and Komuro 1996; Rumessen and Thuneberg 1991). However, there are relatively few reports focusing on the role of caveolae in ICC pacemaking function (Daniel et al. 2009) compared with SMCs. Furthermore, in ICCs, mitochondrial Ca^2+^ uptake is believed to play a crucial role in the generation of pacemaker currents (Sanders 2019). Although the concept of the “Ca^2+^ microdomain” (Rizzuto and Pozzan 2006) and “pacemaker unit” in ICCs (Sanders et al. 2006, 2000)—involving the ER, mitochondria, and plasma membrane—has been proposed based on pharmacological experiments with TEM images, their precise 3D geometry at the nanoscale remains unknown. Detailed morphological information could shed light on new interpretations of several unresolved questions, clarifying the controversial understanding and variety of reactions in physiological and pathological studies (Hwang et al. 2019; Drumm et al. 2018). To address this issue, Focused Ion Beam/Scanning Electron Microscopy (FIB/SEM) analysis was performed. This new method enables the visualisation of 3D ultrastructures in tissue samples without the need for cell isolation (Tamada et al. 2017; 2021). This technique reveals a novel 3D ultrastructure, showing morphological features that are unimaginable with conventional microscopy, such as TEM. Using this advantage, we examined morphological details, focusing on organelle structures, such as the ER/SR and mitochondria, which are involved in Ca^2+^ regulation, in SMCs, ICCs, and PDGFRα+ cells.

Furthermore, FIB/SEM enables quantitative analyses, such as distance and volume measurements, at the ultrastructural level, where conventional TEM is limited (Tamada et al. 2020). In particular, when analysing membrane contact sites, the nanoscale resolution of FIB/SEM serves as a powerful tool for describing these structures as continuous, sheet-like interfaces (Elgendy et al. 2024). Using this advantage, the nature of the microdomains in ICCs was explored to obtain geometric data, such as distances and volumes, thereby enhancing our understanding of the Ca^2+^ regulation mechanisms underlying pacemaker activity.

## Material and methods

The overall workflow is summarised in Supplementary Fig. 1.

### Animals

All animal experiments were conducted in accordance with the Guidelines for Animal Experiments and the Regulations for Animal Research at the University of Fukui. C57BL/6 mice (aged 8–24 weeks) were obtained from SLC (Hamamatsu, Japan) and maintained in our laboratory with ad libitum access to food and water. Male and female mice were used for outline analysis, whereas only male mice were used for sphere analysis.

### FIB/SEM sample preparation

For FIB/SEM sample preparation, the mice were dissected under terminal anaesthesia and euthanised using isoflurane. The stomach was cut open, pinned onto a silicon-coated dish, and immersed in half Karnofsky fixative (containing 2% paraformaldehyde and 2% glutaraldehyde in 0.1 M phosphate buffer, pH 7.4). After 15 min, the specimens were trimmed into small pieces, retaining the entire gut wall from the mucosa to the serosa, and the pieces were placed in the same fixative for 2 h at 4 °C. Specimens were subsequently rinsed in the same buffer and fixed with 1.5% potassium ferrocyanide and 2% osmium tetroxide for 1 h at 4 °C. After rinsing with distilled water, the specimens were treated with 1% thiocarbohydrazide, rinsed with distilled water, treated again with a 2% osmium tetroxide solution for 1 h at room temperature, and washed with distilled water (rOTO method). For en bloc staining, the specimens were immersed in 1% uranyl acetate overnight at room temperature and washed with distilled water. Specimens were stained with Walton’s lead aspartate solution at room temperature (Deerinck et al. 2010). Finally, the samples were dehydrated through a graded ethyl alcohol series and ice-chilled acetone, embedded in epoxy resin (Epon 812), and polymerised at 65 °C for 3 d.

### FIB/SEM observation

The surfaces of the resin-embedded samples mounted on metal stubs were trimmed with a diamond knife until the circular, myenteric plexus, and longitudinal muscle layers were exposed. A carbon protective layer was then coated onto the exposed surfaces, and the stub was mounted on the FIB/SEM stages (Scios; Thermo Fisher Scientific). After the target cells were identified on the surface by SEM, the milling area was protected by carbon deposition. Serial images of the block face were then acquired through repeated cycles of surface milling using a focused gallium ion beam (FIB) and acquired using SEM as a compositional contrast image from backscattered electrons (FIB parameters: accelerating voltage, 30 kV; current, 1.0 nA; milling step size, 20 nm. SEM parameters: accelerating voltage, 2.0 kV; current, 0.10 nA; dwell time, 3 µs; resolution, 3072 × 2048 for outline visualisation or 6144 × 4096 for quantitative analysis). These repeated cycles were controlled using Auto Slice & View 3G operating software (Thermo Fisher Scientific).

### 3D image reconstruction

The 3D images were reconstructed using Z-stacked X–Y serial section images segmented manually for each targeted element, such as the plasma membrane (PM), ER, and mitochondria, MAM contacts, using Amira 3D visualisation software (Thermo Fisher Scientific).

### Geometrical analysis

To perform spherical cropped analysis of these geometrical parameters, three caveolae were randomly selected as the centre of the sphere in one cell from three animals (eight-week-old males, C57BL/6).

The volume and surface area of the reconstructed images were measured using the Amira software. To determine the distance between the ER and PM, the average surface distance (ASD) was calculated from every vertex of the polygon mesh in the reconstructed PM structure to the vertex of the polygon mesh in the reconstructed ER as follows:

Surface distance was measured as the distance from the PM surface to the ER. When *S(ER)* denotes the set of vertices of the polygon mesh of ER, the shortest distance between an arbitrary vertex of $PM$ and *S(ER)* is defined as

$$d(PM,S(ER))=\underset{{s}_{ER}\in S(ER)}{min}\| PM-{s}_{ER}\|$$

where $\| .\|$ denoted the Euclidean distance. The ASD is then given by

$$\mathit{ASD}(PM,ER)=\frac{1}{\mid S(PM)\mid }(\sum_{{s}_{PM}\in S(PM)}d(PM,S(ER)).$$

Finally, the average distance of the ASD from the nine spheres was calculated to determine the trend of the distance between the ER and PM.

To measure mitochondria-associated membrane (MAM) contact, the surface areas were calculated after segmenting the MAM structure and reconstructing in spherical regions.

The coefficient of variation was also calculated with each measured number as follows:

$$\mathrm{Coefficient}\;\mathrm{of}\;\mathrm{variation}\;(\mathrm{CV})\:=\:\mathrm{standard}\;\mathrm{deviation}/\mathrm{sample}\;\mathrm{mean}$$

## Results

### Overview of ER/SR structures and mitochondria distribution in smooth muscle cells, ICCs, and PDGFRα+ cells

**Fig. 1:**
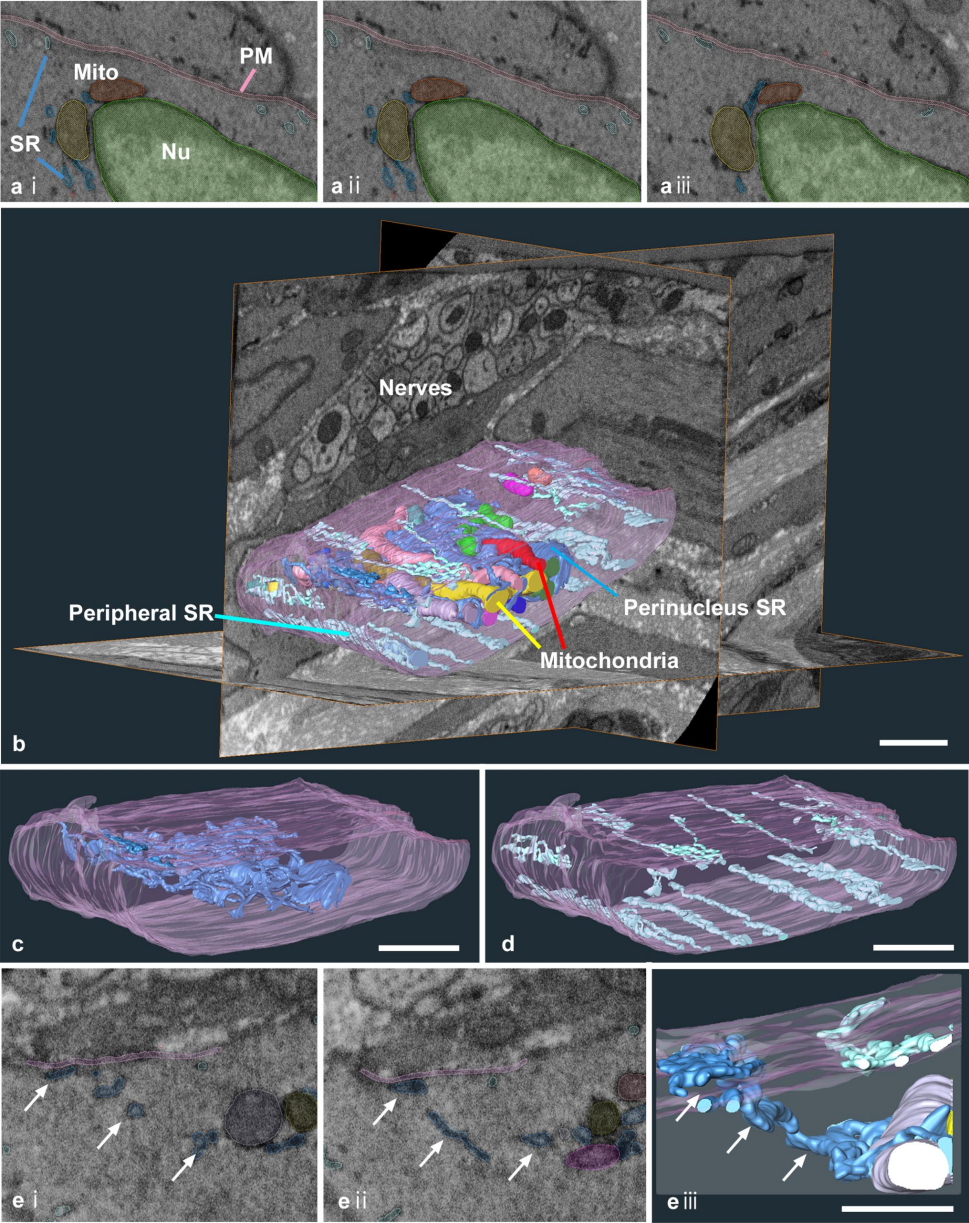
SR structure in the smooth muscle cells (SMC). **a** The representative serial images show manual segmentations of targeted elements, like PM (segmented with pink), SR (blue), mitochondria (orange or yellow), and nuclei (green) in the SMC. **b** Representative reconstructed SR (blue) and mitochondria (coloured) images in SMC with stacking of several hundred segmented images are shown in **a**, in the region containing peripheral cells such as nerves and other SMCs. It could be observed that SR was separated into peripheral (light blue) and perinuclear ones (dark blue). The PM is coloured in pink. **c** The higher magnification image of **b** shows only the perinuclear SR without showing mitochondria. **d** The higher magnification image of **b** shows only the peripheral SR, forming a line-like structure extending along the longitudinal axis of SMC. **e** The serial images show the connecting part between peripheral and perinuclear SR (i and ii) and the reconstructed image (iii). The arrows indicate continuous parts of the SR. Plasma membrane, pink; SR, blue; each mitochondrion, coloured. Scale bar: **b–d **1 µm; **e** 500 nm

To understand how the 3D ultrastructure revealed by FIB/SEM characterises organelle structures in gut motility elements, low-magnification overviews were obtained for SMCs, PDGFRα+ cells, and ICCs in the gastric muscle layer of mice. The 3D reconstructed images of SMC after segmentation (Fig. 1a and b) showed that SR distributions can be broadly classified into two subtypes: perinuclear SR (Fig. 1c) and peripheral SR (Fig. 1d). The peripheral SR extends along the longitudinal axis of the smooth muscle, is located beneath the plasma membrane, and forms a line-like structure. Although several peripheral SR lines exist along the PM, they are rarely connected. In contrast, the perinuclear SR exhibits a 3D arrangement, forming a complex mesh-like structure around the mitochondria. The morphology of the perinuclear SR resembled the typical ER distribution observed in other cells, such as neurones. Although peripheral and perinuclear SR were not directly connected, a small connecting structure was detected (Fig. 1e). The mitochondria aggregated in the inner cytoplasm and were sparsely distributed near the PM (Figs. 1b and 3a).

**Fig. 2:**
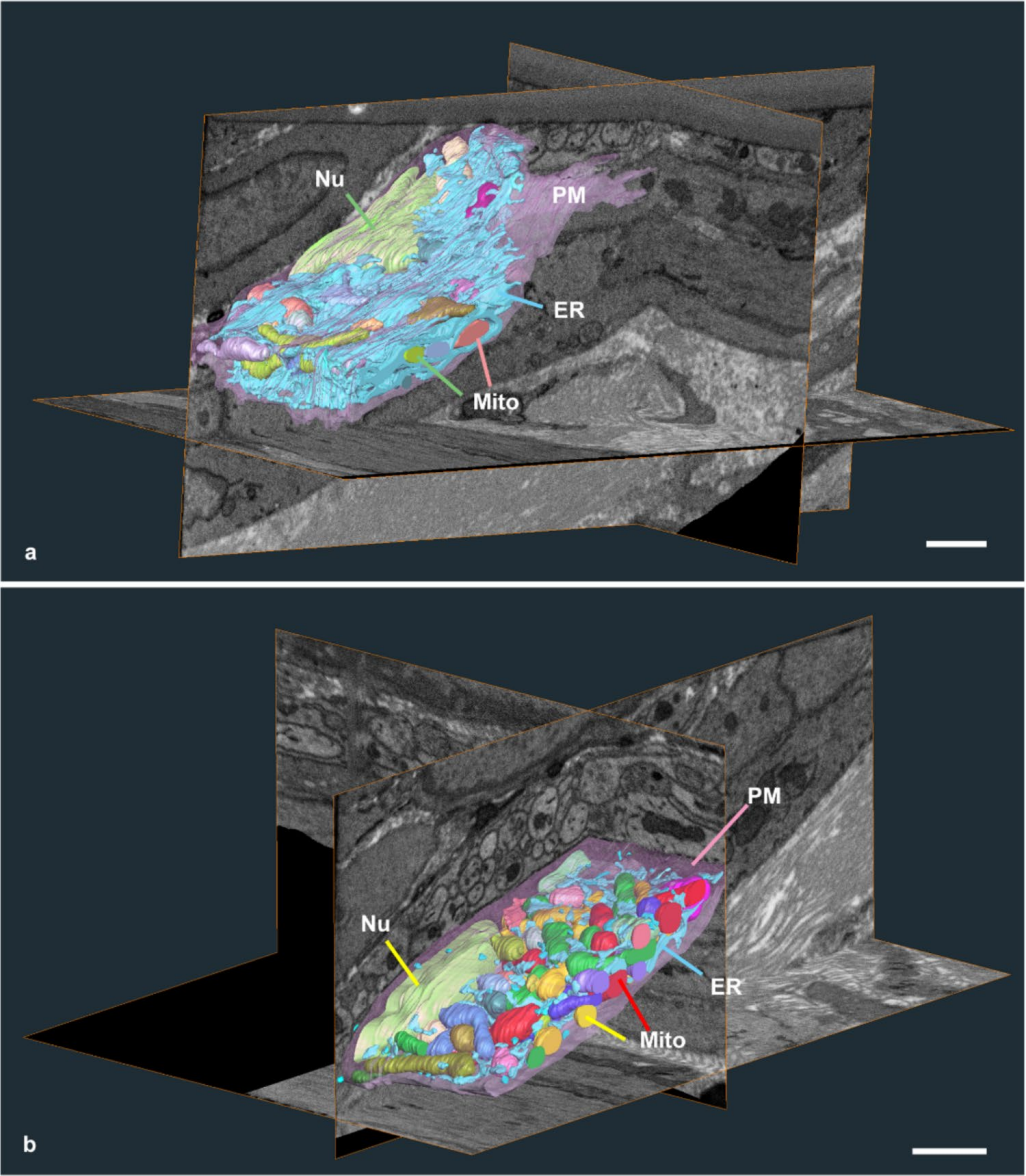
ER structure in the PDGFRα+ cells and the interstitial cells of Cajal (ICCs) distributed around nerve bundles in the muscle layer. **a** A representative reconstructed image of ER (blue) and mitochondria (coloured) in the part containing the nucleus (green) in the PDGFRα+ cells in the region with peripheral cells. The sheet-like ER is distributed throughout the cytoplasm. **b** Representative reconstructed images of the ER (blue) and mitochondria (coloured) in the region containing the nucleus (green) in the ICC. Plasma membrane, pink; and nucleus, green. Scale bar: 1 µm

Next, ICCs and PDGFRα+ cells were examined as stomach motility coordinators. Using conventional TEM, these cells can be distinguished based on several features, including the degree of ER and mitochondrial abundance, cell process structures, and cytoplasmic tone (Komuro et al. 1999; Iino et al. 2009). Furthermore, in the muscle layer, ICCs and PDGFRα⁺ cells are typically arranged around nerve bundles. Using these criteria, they were identified in the muscle layer, and serial image stacks were acquired. The 3D reconstructed images after segmentation revealed sheet-like structures with abundant ER throughout the cytoplasm of PDGFRα+ cells, within which most mitochondria were embedded (Fig. 2a). In contrast, although ICCs also contained relatively abundant ER, their 3D structures showed slightly distinct features: instead of sheet-like structures, they exhibited cytoplasmic mesh-like structures with abundant mitochondria (Fig. 2b), enabling the visualisation of mitochondria embedded within the ER. Although ER abundance was anticipated, the novel 3D imaging revealed not only this abundance but also structural heterogeneity of the ER between PDGFRα⁺ cells and ICCs.

In short, the overview analysis with FIB/SEM revealed new ER/SR morphological details, such as line-like structures in SMCs, sheet-like structures in PDGFRα⁺ cells, and mesh-like structures in ICCs.

### The microdomain with ER/SR and mitochondria around caveolae

To gain new insights into the Ca^2+^ concentration regulatory system in SMC and ICCs, we examined the microdomain structures of the ER/SR, PM, and mitochondria. Caveolae on the PM were set as the centre of Ca^2+^ spark activity, and a spherical region with a radius of 400 nm surrounding each caveola was defined as the microdomain, referring to the number reported in SMC research describing hotspots of local Ca^2+^ flux (Duan et al. 2019; Saeki et al. 2019).

**Fig. 3:**
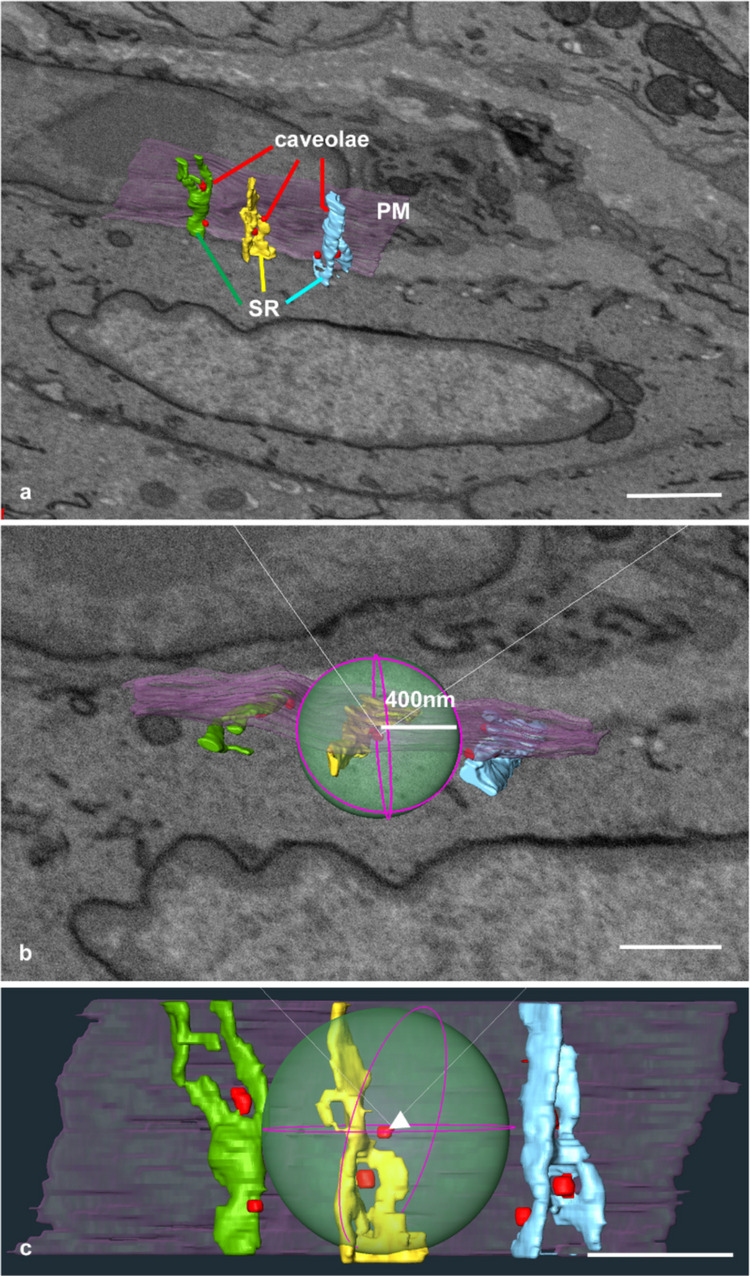
Sphere analysis for SMC. **a** Outline of three representative reconstructed images for the SR (green, yellow, blue), PM (pink), and caveolae (red) in the SMC. **b** The cropped images with sphere regions (green) with a radius of 400 nm from the caveolae. **c** Higher magnification of **b** shows representative sphere-cropped images of the ER surrounding one caveolae set as the centre (red, arrowhead). Plasma membrane, pink; SR, blue; yellow, green; caveolae, red. Scale bar: **a** 1 µm; **b, c** 500 nm

Following the 3D reconstruction (Fig. 3a), the defined spherical regions were trimmed (Fig. 3b). As described in the previous section, SMCs exhibited a line-like peripheral ER distribution. Within the caveolae-focused microdomains, the peripheral ER extends across or encircles the caveolae in a circular arrangement (Fig. 3c). Because the mitochondria in SMCs tend to aggregate in the inner site around the nucleus (Fig. 1a and b), the defined spherical region rarely contains mitochondria.

**Fig. 4:**
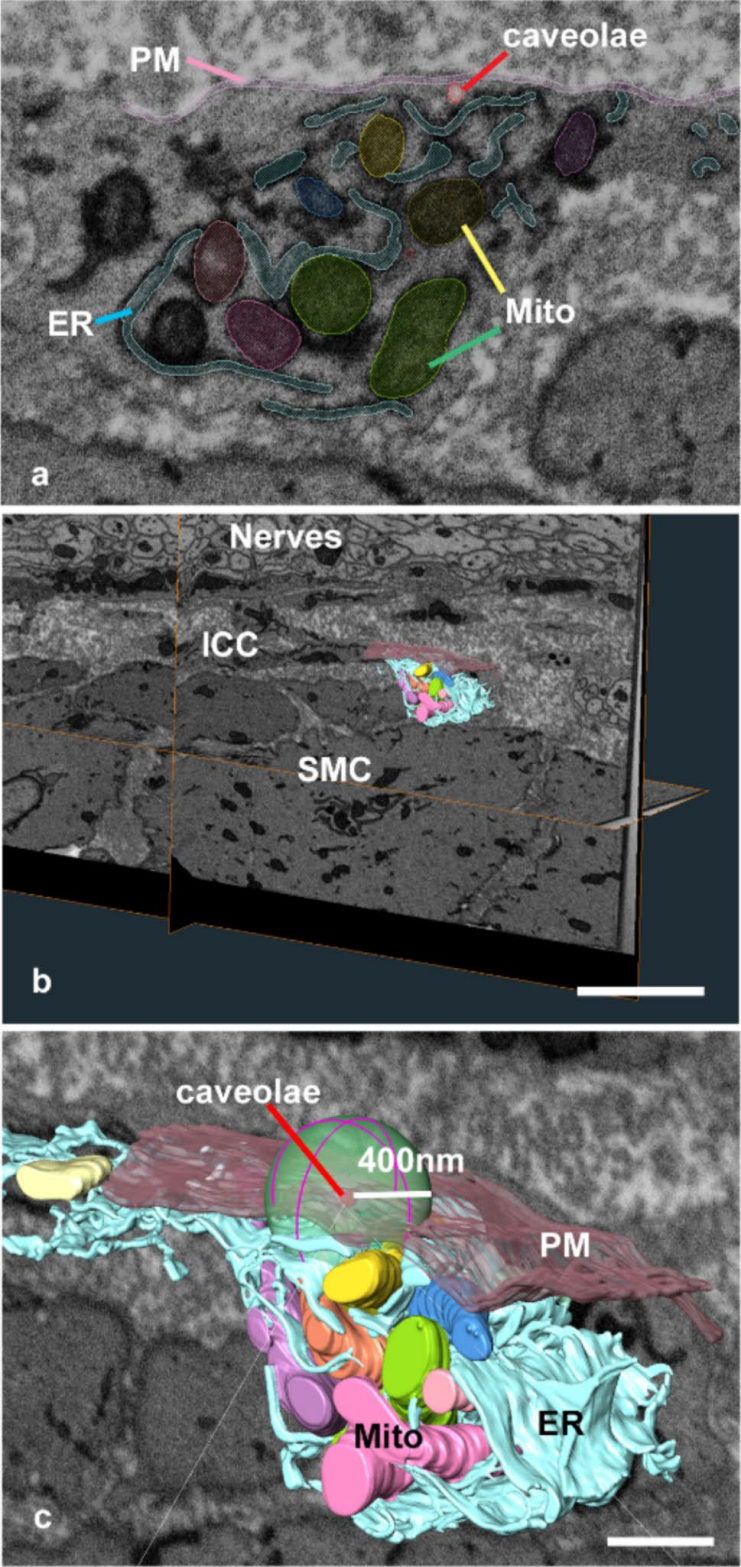
Scheme of Sphere analysis for ICCs. **a** Representative single image with segmentation of the three-dimensional reconstructed images in **b** and **c**. **b** Representative whole-electron microscopy image containing part of the reconstructed area of the PM (pink), ER (blue), and mitochondria (coloured) in the ICC distributed in the myenteric plexus. **c** A higher magnification of the area shows a sphere with a radius of 400 nm from the caveolae set as the centre. Plasma membrane, pink; ER, blue; mitochondria, coloured; and caveolae, red. Scale bar: **b** 3 µm; **c** 500 nm

**Fig. 5:**
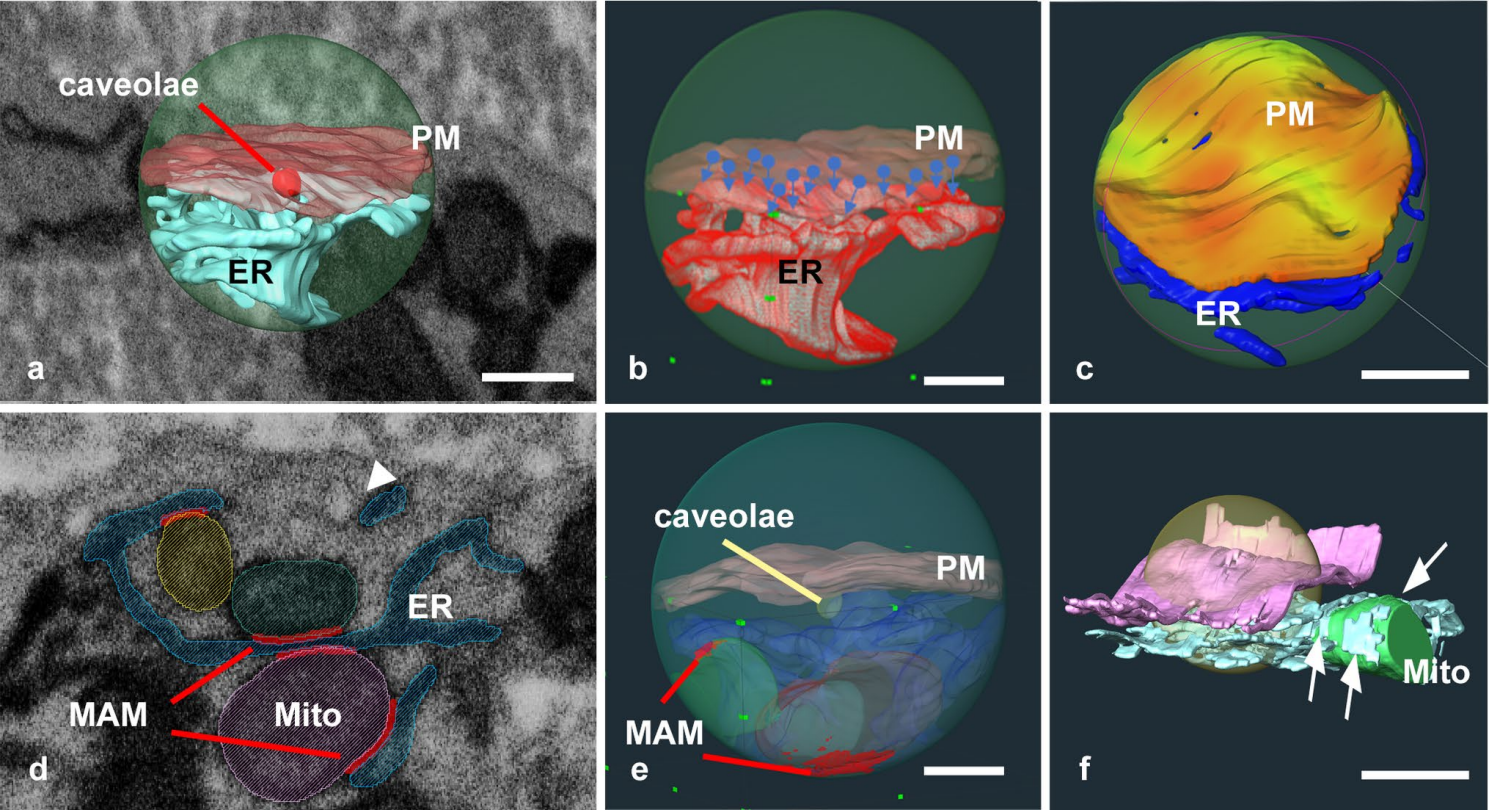
Sphere analysis for ICCs showing distance analysis for ER and PM interaction and MAM surface. **a** PM (pink) and ER (blue) reconstructed images cropped with a 400 nm sphere (green) around caveolae (red). **b** A scheme explaining how to measure the distance between the ER and PM. Circles were set on each point in the formed polygon mesh of the PM, and the closest points in the polygon mesh of the ER (arrowheads) from each PM point were measured. **c** Distance results are shown as a heatmap. A warmer tone indicates a close interaction (small distance), and a cooler tone indicates a far interaction (large distance). **d** The representative single images with segmented MAM in the red line. The structures segmented with blue are the ER, and mitochondria are segmented in yellow, green, and purple. Arrowheads indicate the point where ER is localised extremely close to caveolae. **e** Reconstructed and sphere-cropped images shown in **d**. By stacking the segmented series sections as shown, MAM sheet-like structures were obtained as indicated in red. **f** Existence of MAM contact (arrows) of the contiguous ER outer region of the sphere for 400 nm in radius. PM, pink; ER, blue; mitochondria, green; sphere, yellow. Scale bar: **a** 300 nm; **b** 200 nm; **c** 200 nm; **e** 200 nm; **f** 500 nm

In contrast, ICC microdomains around the caveolae contain abundant ER and mitochondria. For the microdomain analysis, ICCs in the myenteric plexus (ICC-MY) distributed in the antrum were examined as representative pacemaker cells. After segmentation (Fig. 4a) and reconstruction (Fig. 4b), a 400 nm spherical region was defined for geometrical analysis using the same criteria applied to SMCs (Fig. 4c). The surface area and volume of the reconstructed images were measured using Amira software. Despite high variability, the ER volume within the spheres averaged 13.0 × 10^6^ ± 5.1 × 10^6^ nm^3^ (median: 11.0 × 10^6^ nm^3^; spherical region *n* = 9; three spheres were selected from one cell on each animal; animal *n* = 3; 8-week-old male mice) (Fig. 5a). The distance between the ER and PM was measured and calculated using a large number of vertices in the polygonal mesh of the ER and PM using the formula described in the materials and methods (Fig. 5b and c). As a result, the mean closest distance connection between ER and PM was 107.0 ± 20.41 nm (spherical region *n* = 9; three spheres were selected from one cell on each animal; animal *n* = 3, 8-week-old male mice). The small average distance suggests that the ER was continuously located near the PM. In particular, when only caveolae were focused on, an extremely close ER distribution of less than 20 nm was observed locally (Fig. 5d, arrowheads).

Mitochondria-associated membranes (MAMs) are essential for Ca^2+^ uptake (Pizzo et al. 2012), and MAM contacts were observed in this study. The MAM contact was designated as a contact structure of less than 20 nm between the mitochondria and ER, and the corresponding surface area was analysed (Fig. 5d and e). The analysis of the spheres was the same as that used for the ER-PM analysis described above, and some spheres did not have mitochondria in the restricted region (spheres with mitochondria, 5/9; spheres without mitochondria, 4/9). As the spheres without mitochondria around the caveolae showed no MAM, these regions were omitted from the surface area analysis. As a result, the mean MAM contact amongst the MAM-positive sphere was 54 × 10^3^ ± 29 × 10^3^ nm^2^, and the median was 53 × 10^3^ nm^2^, although the variability was much higher than that in ER-PM distance analysis (coefficient of variation in ER and PM distance, 0.19; in MAM contact, 0.53). Because of the consecutive ER structures in the volume, even the sphere without mitochondria tended to have MAM just beyond the 400 nm border (Fig. 5f). Although the influence and mechanism of Ca^2+^ diffusion in the ER were not considered in this study, it is assumed that the nearby MAM broadly influences all ER around the caveolae. Furthermore, although several caveolae were aggregated in the spherically cropped region, they were not considered in the analysis. If the central caveolae are shifted slightly in the vicinity, they are highly likely to possess mitochondrial and MAM structures in a restricted region. 

## Discussion

In this study, FIB/SEM analysis revealed the detailed 3D structure of the ER distribution, primarily in the components that regulate gut motility. These reconstructions revealed new aspects that could not be detected in two-dimensional (2D) images and indicated significant differences among them. For example, although both ICCs and PDGFRα⁺ cells use Ca^2+^-dependent conductance for their function, the 3D images described markedly different patterns of distribution. The findings suggested that functional differences amongst cells should be explained not only by their proteins or channels’ expression but also by the geometrical circumstances for their functions. Furthermore, although both ICCs and SMCs possess caveolae on the PM, their spatial relationships with ER/SR differ markedly. The 3D imaging also revealed that the ER in ICCs is a more continuous organelle than that in SMCs. This suggests that ICCs may employ a more complex Ca^2+^ diffusion mechanism, involving molecular dynamics across an extensively widespread ER network.

Additionally, the study demonstrated that the reconstructed images enabled analysis of the distance between two membrane structures, specifically, the ER–PM and MAM contacts. The ER–PM distance in ICCs was found to be approximately 100 nm, a value that can be used to discuss physiological functions, although the dispersion was greater than expected. Furthermore, geometrical information related to mitochondria, including MAMs, appeared to depend significantly on specific cellular locations. These diversities, explored by precise 3D ultrastructural analysis, provide deeper insights into their physiological features and a new aspect to accelerate functional analysis.

Although a more precise regional definition and sufficient data collection might indicate more uniform geometric data, area-specific microdomains should be considered to elucidate the physiological mechanisms in ICCs more accurately. For instance, in ICCs or ICC-like cells, which have been reported to generate spontaneous Ca^2+^ elevations, the Ca^2+^ release typically originates from some specific sites and subsequently propagates throughout the entire cell (Johnston et al. 2005; Hashitani et al. 2010; Drumm et al. 2017; Gupta et al. 2024). A comparison between the release site of the Ca^2+^ and other regions may be necessary in future studies to obtain more physiologically relevant geometric data. Furthermore, although only ICC-MY was examined as an initial step in this geometrical analysis, focusing on the functional differentiation among ICC subtypes—such as ICC-MY versus ICC distributed in the muscle layer (ICC-IM) and ICC-MY in the stomach versus in the small intestine—may also reveal a clear relationship between function and morphology.

The responses to antagonists of Ano1, Ca^2+^-activated chloride channels, and mitochondrial function inhibitors have been reported to vary among ICC subtypes (Sanders 2019; Hwang et al. 2009; Strege et al. 2015; Sung et al. 2016). These variations have previously been explained by some interpretations, such as non-specific effects (Drumm et al. 2018). However, the ultrastructural arrangement described in this study may represent another factor that could account for the differences in pharmacological reactions. The interactions between them at the ultrastructural level may determine their physiological reactions in addition to the protein expression levels or total volume of certain elements. Furthermore, the pharmacological analyses of tissues may reflect the combined effects of several components. From this perspective, morphological analyses targeting the information of each cell are important to accurately interpret their functions.

In this study, a spherical region 400 nm in radius from the centred caveolae was used for geometrical analysis. Although this value appears reasonable for smooth muscle cells (Duan et al. 2019; Saeki et al. 2019), it remains to be determined which value is most appropriate for ICCs, as a 2 µm × 0.75 nm area was used in 2D simulations of ICC pace making (Means and Cheng 2014). Several studies have attempted to explore the complex ICC pacemaker functions using mathematical approaches (Means and Cheng 2014; Lees-Green et al. 2014; Corrias and Buist 2008; Faville et al. 2008). Although geometric data have been adopted for these analyses, in addition to the ion and electrical parameters, they have thus far been derived from 2D images. The FIB/SEM 3D quantitative data presented in this study are expected to facilitate the development of more realistic simulation tools.

The spherical regions analysed in ICCs showed a clear MAM structure, and some buffering systems appeared to be present within the microdomains of the ICCs’ pacemaker. Although it has been reported that a distance of approximately 50 nm between the ER and mitochondria or between the PM and mitochondria is sufficient for Ca^2+^ buffering (Cartes-Saavedra et al. 2025), the attachment provided by MAM structural contacts is essential for the function of the mitochondrial Ca^2+^ uniporter (MCU) (Pizzo et al. 2012; Rizzuto and Pozzan 2006). On the other hand, because continuous ER structures were also observed in this study, Ca^2+^ diffusion mechanisms in the ER may be considered.

In SMCs, caveolae have been reported to play several crucial roles in Ca^2+^ regulation, such as modulating calcium- and voltage-gated large conductance potassium (BK) channels and Cav1.2, which are activated by Ca^2+^ release from the SR to regulate membrane excitation (Suzuki et al. 2013). SMCs exhibit a compartmentalised arrangement of caveolae and close spatial interactions between the PM and the SR (Akin et al. 2023). Although previous studies have conducted precise geometrical measurements of caveolae, peripheral SR, and mitochondria in SMC (Popescu et al. 2006), as well as 3D analyses (Tong et al. 2009), our observation of a distinct line-like structure without connection to those on either side of the peripheral ER is a relatively novel finding, enabled by the “volume analysis” of approximately 10^3^μm^3^, with several hundreds of serial images using FIB/SEM.

In this study, ICC caveolae were also temporally assumed to be representative sites of Ca^2+^ hotspot activity. Some initial Ca^2+^-regulating components co-localise with caveolin-1 in ICCs (Cho and Daniel 2005), and it has been hypothesised that close interactions between caveolae and the ER may account for calcium recycling through L-type Ca^2+^ channels in ICCs (Daniel et al. 2009).

Although there is no clear evidence yet confirming that Ano1 distribution matches that of caveolae, immunohistochemistry of Ano1 in ICCs has been widely reported, and its location appears uniform around the ICC plasma membrane. Ano1 has also been proposed as a potential tethering factor for “ER–PM contact sites,” similar to junctophilin and STIM1 (Lin et al. 2024), and ICCs possess the STIM/Orai mechanism for pacemaker activity (Zheng et al. 2018). In general, STIM/Orai interactions are representative tethering components of ER–PM contacts, forming junctions of less than 20 nm (Luik and Lewis 2007; Wu et al. 2006; Prakriya and Lewis 2015). In the detailed ultrastructural analysis of ICCs using FIB/SEM, most ER–PM interactions did not appear as typical sheet-like structures with contacts less than 20 nm, which were frequently described in our previous study (Elgendy et al. 2024). However, small points of ER–PM contact within 20 nm were detected, particularly just beneath the caveolae. These findings may provide new insights into the STIM/Orai mechanism in the pacemaker activity of ICCs.

There are some limitations regarding the influence of fixation on the ER structure or membrane contacts, and high-pressure freezing may need to be considered. However, under the present tissue preparation conditions, MAM was observed, and ER–PM contact was also detected in other tissues (Elgendy et al. 2024). Furthermore, if a comparison among any cell type is performed in the future, even within the same sample, these morphological findings should be sufficiently reliable for discussion.

This novel morphological approach, revealing 3D ultrastructure, provides new insights into the mechanisms underlying gut motility. In particular, FIB/SEM appears to be a powerful tool for achieving the sufficient resolution required to investigate crucial membrane contacts and perform detailed volume analyses, which are not feasible with conventional TEM analysis.

## Supplementary Information

Below is the link to the electronic supplementary material.

ESM 1 (JPG 494 KB) Figure 1

ESM 2 (DOCX 15.4 KB) Figure 1 Legend

## Data Availability

All relevant data are available from the corresponding authors.
