# Correction to: ER morphological analysis associated with interstitial cells of Cajal and smooth muscle cells in the murine stomach

**DOI:** 10.1007/s00441-025-04026-5

**Published:** 2025-11-10

**Authors:** Hiromi Tamada, Satoshi Iino

**Affiliations:** Anatomy, Graduate School of Medical Sciences, University of Fukui, 23‑3 Matsuokashimoaizuki, Yoshida‑Gun, Eiheiji‑cho, Fukui, 910‑1193 Japan

**Correction to: Cell and Tissue Research**

10.1007/s00441-025-04016-7

The authors regret that the text that reads:

In this study, a spherical region 400 nm in **diameter** from the centred caveolae was used for geometrical analysis.

In page 8, under the “Discussion” section of the original published article is incorrect. It should read as below:

In this study, a spherical region 400 nm in **radius** from the centred caveolae was used for geometrical analysis.

The original article has been corrected.

