# Supplementary figures and images for: ER morphological analysis associated with interstitial cells of Cajal and smooth muscle cells in the murine stomach
Source: Cell Tissue Res. 2025 Oct 28;402(3):333–44. doi: 10.1007/s00441-025-04016-7 (PMC12727777; doi:10.1007/s00441-025-04016-7)

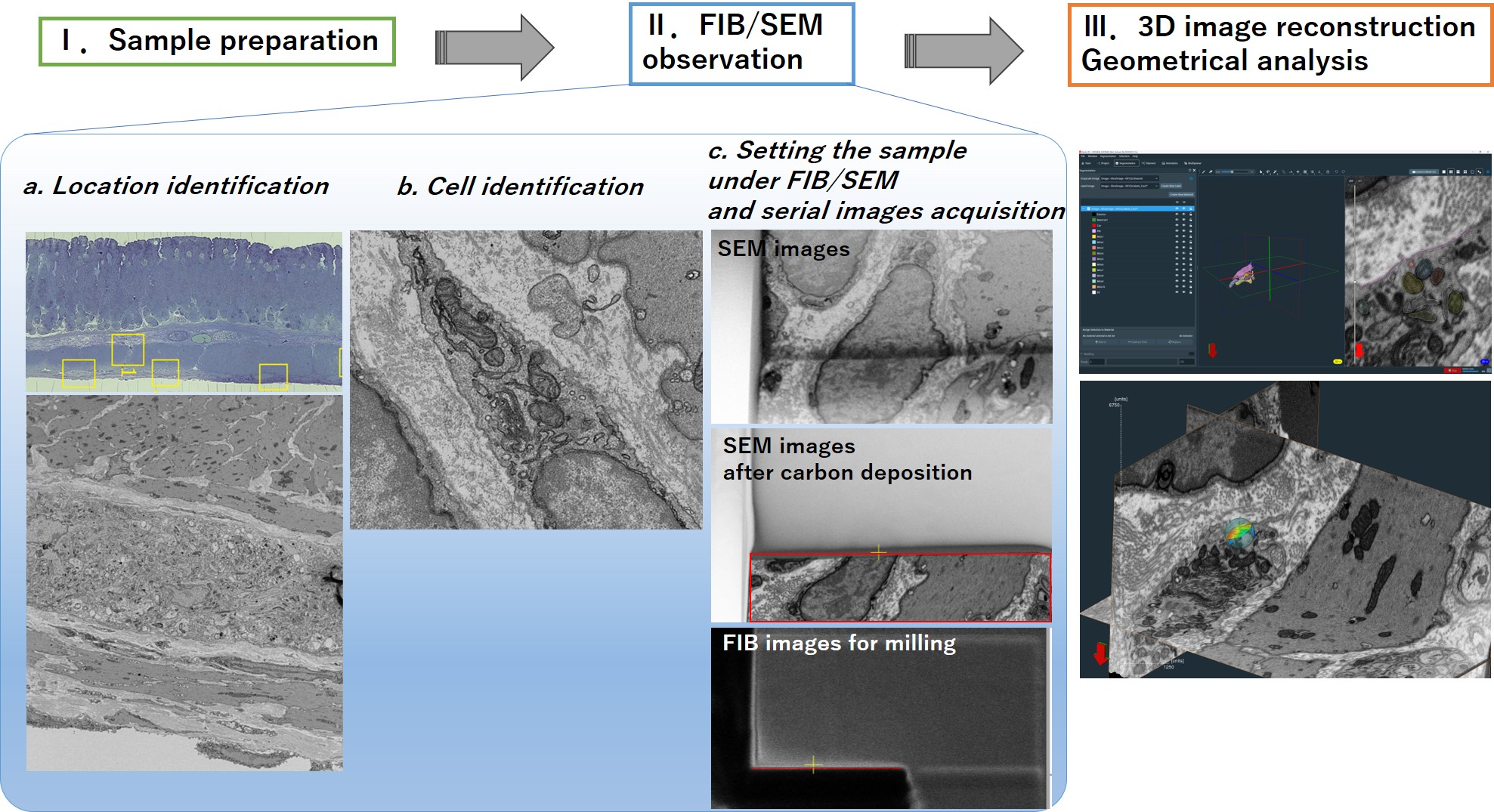

Supplement: Supplementary file 1 — (JPG 494 KB) Figure 1 [file 441_2025_4016_MOESM1_ESM.jpg]
