# Supplementary material for: ER morphological analysis associated with interstitial cells of Cajal and smooth muscle cells in the murine stomach
Source: Cell Tissue Res. 2025 Oct 28;402(3):333–44. doi: 10.1007/s00441-025-04016-7 (PMC12727777; doi:10.1007/s00441-025-04016-7)
Supplement: Supplementary file 2 — (DOCX 15.4 KB) Figure 1 Legend [file 441_2025_4016_MOESM2_ESM.docx]

**Supplementary Figure 1. Analysis workflow**

**Ⅰ.** Sample preparation is performed as described in the Materials and Methods.

**Ⅱ. a.** After attaching the FIB/SEM sample to the metal tab, the sample surface, from mucosal to the serosal layer, is exposed, and every layer is detected. Upper: Toluidine blue-stained image of the sample surface observed by light microscopy; Lower: the sample surface observed by electron microscopy.

**b.** Furthermore, cells are identified using criteria, such as caveolae and cytoplasmic tone.

**c.** After determining the target, the samples set in the FIB/SEM. Trenches are created around the observation area, carbon was deposited to protect the sample, and the FIB repeatedly milled the observed surface. Upper: SEM images of the observation target, Middle: Carbon-deposited sample; red square indicates the SEM scanning plane for image acquisition, Bottom: FIB images for milling; red line indicates the stat line for milling.

**Ⅲ.** Three-dimensional reconstructions are created by stacking manually segmented serial images (upper panel). After obtaining the reconstructed images, a geometrical analysis is performed (lower panel).
